# Metabolic reprogramming of the retinal pigment epithelium by cytokines associated with age-related macular degeneration

**DOI:** 10.1042/BSR20231904

**Published:** 2024-04-16

**Authors:** David S. Hansman, Yuefang Ma, Daniel Thomas, Justine R. Smith, Robert J. Casson, Daniel J. Peet

**Affiliations:** 1 School of Biological Sciences, University of Adelaide, Adelaide, SA, Australia; 2 College of Medicine and Public Health, Flinders University, Adelaide, SA, Australia; 3 South Australian Health and Medical Research Institute (SAHMRI), Adelaide Medical School, University of Adelaide, Adelaide, SA, Australia; 4 Discipline of Ophthalmology and Visual Science, Adelaide Medical School, University of Adelaide, Adelaide, SA, Australia

**Keywords:** carbohydrate metabolism, cytokines, glycolysis, inflammation, retinal degeneration, retinal pigment epithelium

## Abstract

The complex metabolic relationship between the retinal pigment epithelium (RPE) and photoreceptors is essential for maintaining retinal health. Recent evidence indicates the RPE acts as an adjacent lactate sink, suppressing glycolysis in the epithelium in order to maximize glycolysis in the photoreceptors. Dysregulated metabolism within the RPE has been implicated in the pathogenesis of age-related macular degeneration (AMD), a leading cause of vision loss. In the present study, we investigate the effects of four cytokines associated with AMD, TNFα, TGF-β2, IL-6, and IL-1β, as well as a cocktail containing all four cytokines, on RPE metabolism using ARPE-19 cells, primary human RPE cells, and *ex vivo* rat eyecups. Strikingly, we found cytokine-specific changes in numerous metabolic markers including lactate production, glucose consumption, extracellular acidification rate, and oxygen consumption rate accompanied by increases in total mitochondrial volume and ATP production. Together, all four cytokines could potently override the constitutive suppression of glycolysis in the RPE, through a mechanism independent of PI3K/AKT, MEK/ERK, or NF-κB. Finally, we observed changes in glycolytic gene expression with cytokine treatment, including in lactate dehydrogenase subunit and glucose transporter expression. Our findings provide new insights into the metabolic changes in the RPE under inflammatory conditions and highlight potential therapeutic targets for AMD.

## Introduction

The complex metabolic relationship between the cells of the outer retina, particularly the retinal pigment epithelium (RPE) and photoreceptors, has recently garnered renewed interest due to its potential implications in health and disease. At the center of these interactions is the unusual metabolic phenomenon known as aerobic glycolysis or the ‘Warburg effect’. Otto Warburg’s investigations in the 1920s first identified the retina as exhibiting this process in which most glucose carbons are metabolized into lactate, rather than taken up to fuel mitochondrial oxidative phosphorylation (OXPHOS), despite the presence of sufficient oxygen [1].

The RPE is a monolayer of cells that blankets the outer retina, located between Bruch's membrane and the underlying choroid and the intercalated photoreceptors, playing several key roles that sustain retinal homeostasis. Its functions support retinal physiology by maintaining and regulating transport across the blood-retina barrier, phagocytosing photoreceptor outer segments, and regenerating visual pigments such as 11-cis-retinal [2–4].

In a landmark study, Kanow et al. demonstrated that lactate suppresses glycolysis in the RPE, permitting the transit of glucose across this monolayer from the choroid to meet the prodigious energy demands of the photoreceptors [5]. The lactate excreted as the waste product of photoreceptor metabolism is then converted to pyruvate in the RPE, serving as a substrate for the tricarboxylic acid (TCA) cycle in the mitochondria and facilitating energy production through OXPHOS. Recent investigations indicate that metabolic interplay between the RPE and photoreceptors extends beyond just glucose and lactate. These cells are now believed to undergo a complex exchange of metabolites including citrate, succinate, glutamate, and aspartate [6–8]. These findings have profoundly impacted our understanding of the retinal metabolic ecosystem, highlighting potential implications for retinal health and disease.

Age-related macular degeneration (AMD) is a multifactorial, progressive generative disorder of the central retina. It is the leading cause of blindness in high-income countries and afflicts an estimated 200 million people globally as of 2020 [9,10]. Recent research has identified dysfunctional retinal metabolism as a key driver of pathology in AMD. The photoreceptors of individuals with AMD have altered mitochondrial dynamics and dysregulated glycolytic gene expression [11,12]. Moreover, RPE cells from AMD donors often exhibit severe mitochondrial defects, including disrupted architecture, diminished count and mass, altered bioenergetics, and accumulations in mitochondrial DNA damage that correlates with disease severity [13–18]. As a result, the RPE is forced to rely more heavily on glycolysis to fulfill its ATP requirements [13].

With the progression of AMD, and aging more generally, the RPE and its surrounding structures – Bruch’s membrane and choroid – become hubs of chronic inflammation in the macular region. This is driven by a confluence of factors including damage-associated molecular patterns (DAMPs), RPE senescence, and extracellular protein and lipid deposits such as drusen [19–21]. As a consequence, the localized levels of inflammatory cytokines such as TNFα, TGF-β, IL-6, and IL-1β rise, driven by infiltrating immune cells and elevated expression in the RPE itself [20].

Although our understanding of cross-talk between inflammation and metabolism is still emerging, these cytokines have been shown to influence glucose metabolism in other tissues and disease models. TNFα increases glycolytic gene expression in porcine Sertoli and human prostate epithelial cells [22,23], and increases both glucose consumption and lactate production in murine fibroblasts [24]. TGF-β signaling promotes glycolytic gene expression in mesothelial cells and glioblastoma cells [25,26], and increases both glucose consumption and lactate production in pancreatic ductal adenocarcinoma cells [27]. IL-6 increases glucose uptake and lactate production in adipocytes [28], skeletal muscle [29], and colorectal cancer cells, increasing a host of glycolytic genes in the latter [30]. IL-1β increases glucose uptake in multiple human cell types including rheumatoid synovial cells, gingival fibroblasts, adipocytes, astrocytes, and chondrocytes [31–35]. These cytokines exert their biological effects through both distinct and shared intracellular signaling pathways including the PI3K/AKT/mTOR, MEK/ERK, NF-κB, Smad, and JAK/STAT3 [36–41] pathways.

The RPE expresses receptors for TNFα, TGF-β, IL-6, and IL-1β [42–45]; however, the degree that each of these cytokines influence glucose metabolism in the RPE, and how they might act in combination, remains unclear. Moreover, the specific molecular pathways through which these cytokines might exert their metabolic effects in the RPE is unknown. Investigating the cross-talk between inflammation and metabolism in the retina may reveal the potential for targeting specific cytokines or their signaling pathways as a therapeutic approach in AMD.

In the present study, we aim to elucidate the impact of inflammatory cytokines on RPE glucose metabolism using a combination of *in vitro* and *ex vivo* models. We find that cytokines increase both lactate production and glucose consumption in the ARPE-19 human retinal epithelial cell line, primary human RPE cells, and rat eyecups, with a cytokine cocktail containing all four cytokines having the most consistent effect. We also observe cytokine-specific changes in oxidative metabolism, with the cytokine cocktail inducing substantial increases in oxygen consumption, while increasing mitochondrial volume and ATP production. Lastly, while the influence of signaling pathways such as PI3K/AKT, MEK/ERK, and NF-κB in mediating these cytokine-induced metabolic effects remains uncertain, cytokines were found to alter the expression of glycolytic genes such as LDHA and GLUT-1. Our investigations may provide new insights into the metabolic alterations in the RPE under inflammatory conditions and their potential implications in AMD pathogenesis.

## Results

### Cytokines increase lactate production and glucose consumption in ARPE-19 cells, primary human RPE and rat eyecups

**Figure 1:**
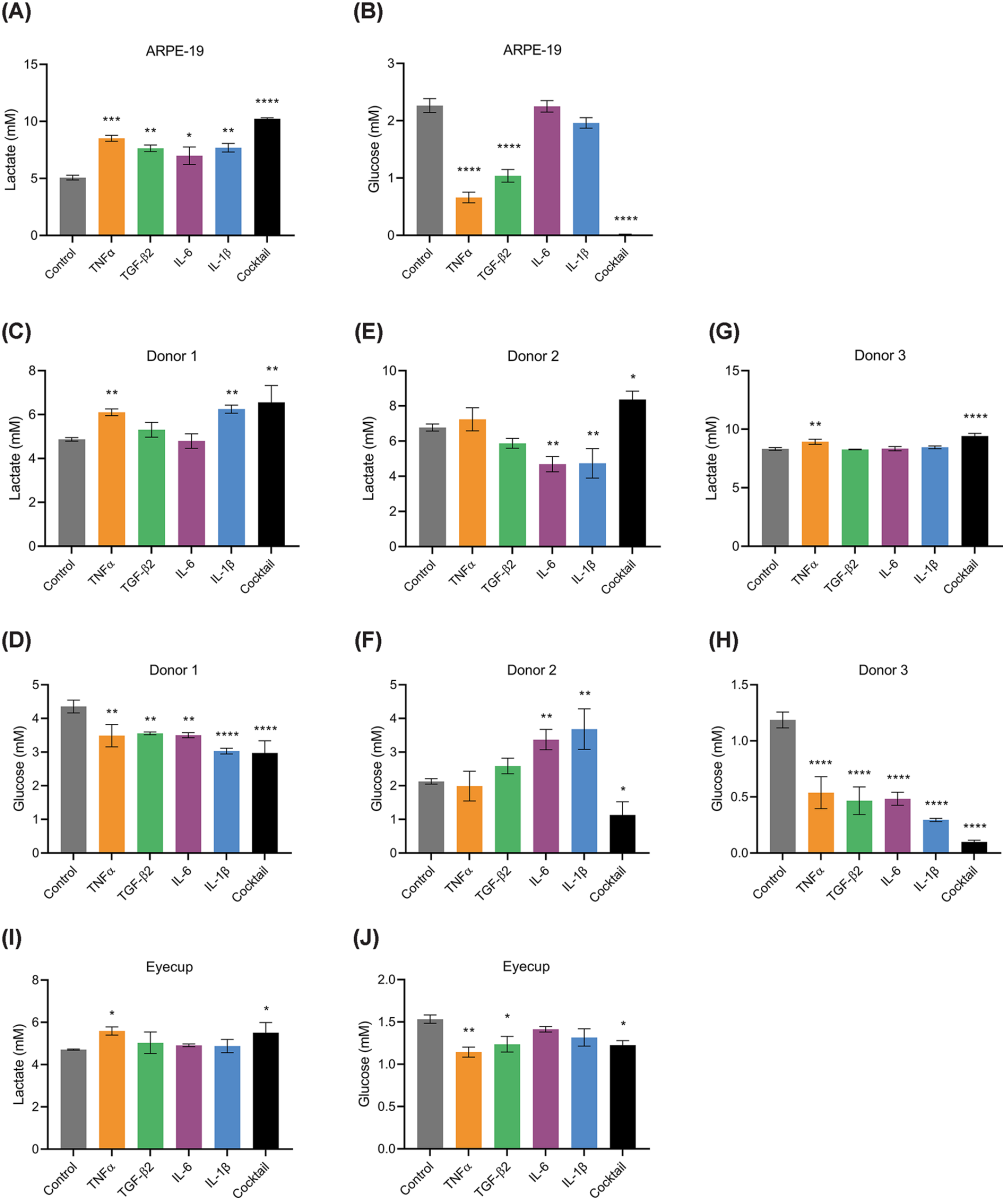
Cytokines increase lactate production and glucose consumption in the RPE The concentration of lactate (**A**) and glucose (**B**) in the culture medium of ARPE-19 cells, measured 24 h after treatment with 20 ng/ml of cytokines (*n* = 3 independent experiments). (**C–H**) Lactate and glucose concentrations in the culture medium of primary RPE cells from three human donors, measured 24 h post-cytokine treatment (20 ng/ml). The concentration of lactate (**I**) and glucose (**J**) in the medium following a 24-h cytokine treatment (20 ng/ml) in rat eyecup cultures (*n* = 3 independent experiments). Error bars show mean ± SEM. Statistical comparisons were performed using one-way ANOVA with Dunnett’s multiple comparisons test against vehicle-treated controls; *P<0.05, **P<0.01, ***P<0.001, ****P<0.0001

To investigate the effects of cytokines on RPE metabolism, we initially analyzed the spontaneously immortalized ARPE-19 cell line, a commonly used RPE model [46,47]. Following a 6- to 8-week differentiation period, the cells were treated with either TNFα, TGF-β2, IL-6, IL-1β, or a combination of these four cytokines (cytokine cocktail) at a concentration of 20 ng/ml in low glucose (5.5 mM) medium for 24 h. Samples of medium were subsequently assayed to measure lactate and glucose concentration. TNFα, TGF-β2, IL-1β, and the cytokine cocktail significantly increased lactate efflux into the medium, with the cocktail eliciting the most potent effect (30% increase, Figure 1A). For most cytokines, these increases in lactate production were mirrored by a decrease in glucose concentration in the medium (Figure 1B). IL-6 and IL-1β however did not significantly decrease the glucose concentration, suggesting they stimulate lactate production using non-glucose carbon sources.

To demonstrate that these results were not specific for the immortalized cell line, primary human RPE cells isolates from eyes of three human donors were also subjected to cytokine treatments for 24 h. We found that the effects of cytokines on lactate production and glucose consumption varied considerably depending on the donor. Specifically, TNFα significantly increased both lactate production and glucose consumption in cell isolates from both donor 1 and donor 3 (Figure 1C, D, G, and H). TGF-β2 also increased glucose consumption in these two isolates without significantly affecting lactate production. The effects of IL-6 and IL-1β also proved to be donor-specific, with these cytokines increasing glucose consumption in the cell isolates from donors 1 and 3 (Figure 1D,H) and decreasing it in the isolate from donor 2 (Figure 1F). However, the cytokine cocktail increased glucose consumption and lactate production across all donors, consistent with the effects observed in the ARPE-19 cells (Figure 1C–H).

Lastly, we validated these results using an *ex vivo* model, treating rat eyecups with cytokines in 5.5 mM DMEM for 24 h. Rodent eyecups have been used elsewhere as an *ex vivo* model to study RPE metabolism [7,48]. After removal of the anterior segment and retina, the eyecup contains the RPE, as well as largely metabolically inert choroid and sclera. Both TNFα and the cytokine cocktail significantly increased lactate production and glucose consumption by approximately 20% (Figure 1I,J). Together, these data demonstrate that while there is some variability between responses with individual cytokines, the cocktail of cytokines consistently increases lactate production and glucose consumption in cultured RPE cells and eyecups *ex vivo*.

Subsequently, we investigated whether the effects of cytokines on lactate production are additive. Differentiated ARPE-19s were treated with varying combinations of cytokines for 24 h before medium samples were assayed for lactate and glucose. Co-treatment of cells with TNFα and TGF-β2 resulted in slightly increased lactate production, while co-treatment of TNFα with either IL-6 or IL-1β resulted in minimal combinatorial effects (Supplementary Figure S1). Addition of IL-6 with any other cytokine had minimal effect, consistent with the observation that IL-6 alone had little effect on lactate efflux. In contrast, TGF-β2 induced substantial increases in lactate efflux when combined with all other cytokines. The maximum effect was observed with the cytokine cocktail, which led to a substantial 70% increase in lactate production (Supplementary Figure S1).

### Cytokines promote both extracellular acidification rate (ECAR) and oxygen consumption rate (OCR) in ARPE-19 cells

To further assess the effects of cytokines on RPE metabolic dynamics, we next quantified the extracellular acidification rate (ECAR) and oxygen consumption rate (OCR). This was achieved using the Seahorse XFe96 analyzer for ARPE-19 cells that had been cultured for 6 weeks in 96-well Seahorse plates. Prior to analysis, the cells were subjected to cytokine treatment at a concentration of 20 ng/ml in 5.5 mM glucose DMEM for 24 h.

**Figure 2:**
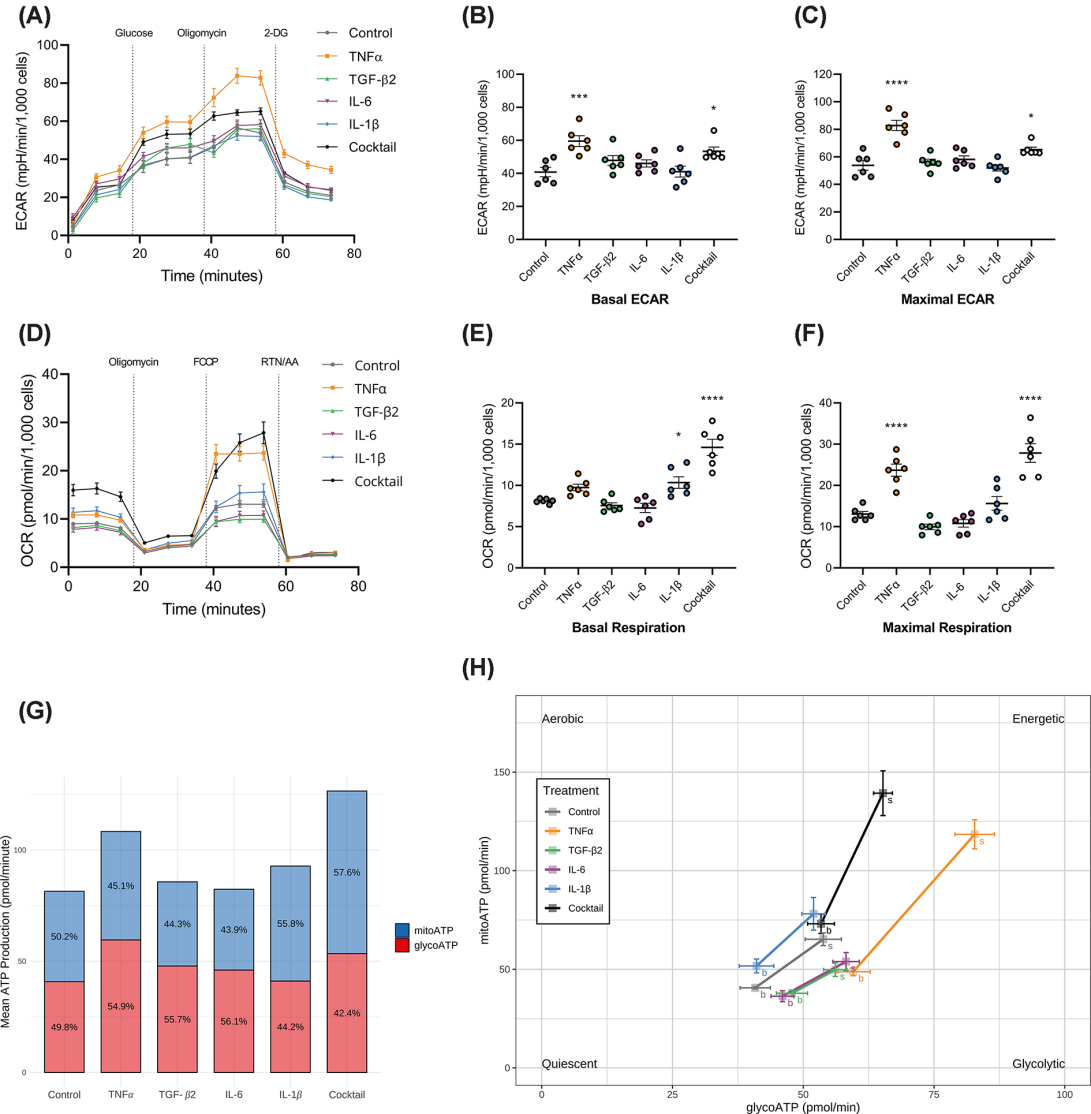
Cytokines influence ARPE-19 bioenergetics ARPE-19 cells were grown in Agilent XFe96 cell culture microplates for 6 weeks to allow for maximum differentiation and treated with 20 ng/ml cytokines for 24 h prior to analysis. (**A**) Real-time measurement of ECAR cytokine-treated ARPE-19 cells, measured with a Seahorse XFe96 Bioanalyzer. Cells were sequentially injected with glucose, oligomycin, and 2-deoxy-D-glucose (2-DG), allowing the measurement of basal glycolysis, glycolytic capacity, and glycolytic reserve, respectively. Plots of the effects of cytokines on (**B**) basal and (**C**) maximal ECAR. (**D**) Real-time measurement of OCR of cytokine-treated ARPE-19 cells, measured with a Seahorse XFe96 Bioanalyzer. Cells were injected sequentially with oligomycin, FCCP, and rotenone/antimycin A to measure the basal, ATP-linked, and maximal respiration, respectively. Plots of the effects of cytokines on (**E**) basal and (**F**) maximal respiration; *N*=6. Error bars show mean ± SEM; *P<0.05, ***P<0.001, ****P<0.0001 determined by one-way ANOVA with Dunnett’s multiple comparison test. (**G**) Bioenergetic profile of ARPE-19 cells following cytokine treatment demonstrating mean mitochondrial and glycolytic ATP production. (**H**) Energy phenotype profile of ARPE-19 cells, showing the mitochondrial and glycolytic ATP production rate for each cytokine treatment under (b) basal and (s) stressed (oligomycin and FCCP-treated) conditions.

The ECAR was measured following glucose and oligomycin injections to obtain the basal glycolytic rate and assess the glycolytic reserve capacity, respectively. Upon oligomycin injection, all cytokine treatments resulted in a slight ECAR elevation (Figure 2A), suggesting that the cells were operating close to their glycolytic capacity, with the exception of the TNFα which generated a more dramatic increase in ECAR. Following TNFα or cytokine cocktail treatment, a significant increase above the untreated control was observed in both basal (Figure 2B) and maximal (Figure 2C) ECAR, corroborating the results from the lactate assays.

To investigate the effects of cytokines on RPE mitochondrial oxidative metabolism, the OCR was measured after injection of oligomycin, FCCP and rotenone/antimycin A, to evaluate the ATP-linked respiration, maximal respiration, and reserve capacity, respectively. Similar to the glycolytic rate, untreated cells demonstrated O_2_ consumption near their capacity under basal conditions (Figure 2D). Treatment with either IL-1β or the cytokine cocktail significantly increased basal respiration (Figure 2E). Additionally, both TNFα and the cytokine cocktail significantly elevated maximal respiration (Figure 2F), consistent with an increase in mitochondrial reserve capacity.

The cytokine cocktail exhibited the largest effects, elevating basal and maximal respiration by 75% and 115%, respectively (Figure 2E,F). Analysis of the bioenergetic profile suggests that TNFα, IL-1β, and the cocktail promote increased basal ATP production, driven primarily by glycolysis with TNFα treatment, and OXPHOS with IL-1β and cocktail treatments (Figure 2G). In response to metabolic stress imposed by inhibition of mitochondrial OXPHOS (oligomycin) and mitochondrial uncoupling (FCCP), ARPE-19 cells undergo a significant shift to more energetic phenotypes following TNFα and cocktail treatments. While both treatments increase aerobic and glycolytic metabolism, TNFα slightly favors glycolysis, whereas the cocktail skews bioenergetics towards aerobic metabolism (Figure 2H).

### Cytokines increase mitochondrial volume and ATP/ADP ratio

**Figure 3:**
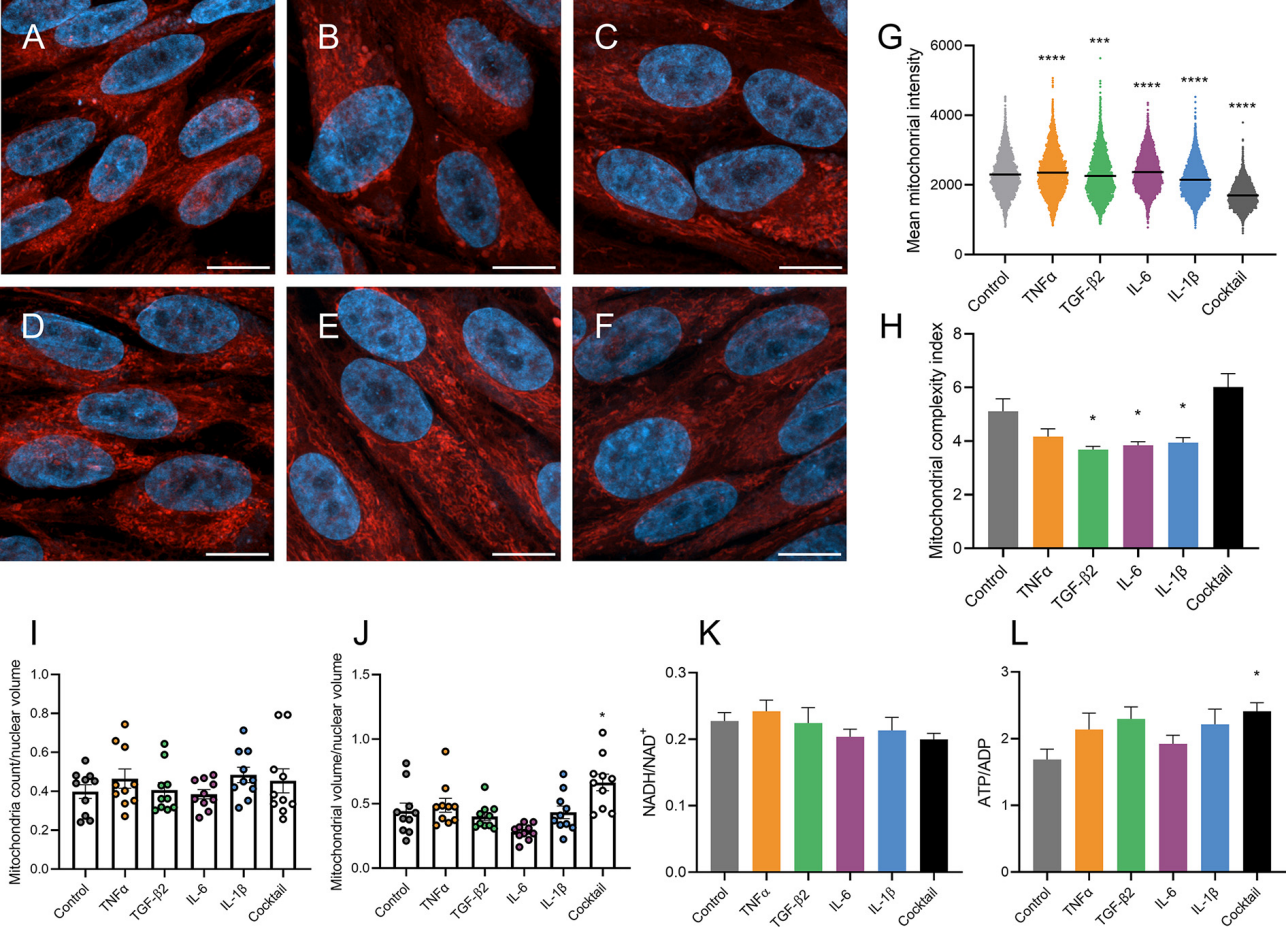
Cytokines influence mitochondrial dynamics (**A–F**) Representative images of differentiated ARPE-19 cells (scale bar = 10 μm), (A) vehicle-treated control, or treated with 20 ng/ml (B) TNFα, (C) TGF-β2, (D) IL-6, (E) IL-1β, (F) cytokine cocktail for 24 h. Images were captured at 40× magnification using a Zeiss LSM 980 Airyscan 2 confocal microscope with mitochondria stained with MitoTracker Red CMXRos (red) and nuclei with DAPI (blue). Confocal Z-stacks were analyzed using Imaris software to determine (**G**) average Mitotracker Red CMXRos staining signal intensity per mitochondrion, (**H**) average mitochondrial complexity index per cell (*n* = 10 cells), (**I**) cellular mitochondria count per nuclear volume (*n* = 10 cells), and (**J**) total cellular mitochondria volume per nuclear volume (*n* = 10 cells). (**K**) NADH/NAD+ ratio was calculated post 24-h cytokine treatment by separately extracting and assaying reduced and oxidized nicotinamide currency metabolites using a cycling method (*n* = 5 independent experiments) (refer to Methods section). (**L**) After 24-h cytokine treatment, ATP/ADP metabolites were extracted from differentiated ARPE-19 cells. ATP was quantified using a luciferase-based assay, and ADP was quantified by converting ADP to ATP in samples and subtracting the unconverted ATP value (*n* = 5 independent experiments). Error bars indicate means ± SEM. Statistical significance: *P<0.05, ***P<0.001, ****P<0.0001, determined by one-way ANOVA with Dunnett’s multiple comparison test.

Given the observed changes in OCR following cytokine treatment, we next determined whether this was accompanied by changes in mitochondrial morphology or dynamics. Differentiated ARPE-19 cells were treated with 20 ng/ml cytokines for 24 h, prior to staining with MitoTracker Red CMXRos and DAPI. Cells were imaged using a Zeiss LSM980 Airyscan 2 confocal microscope (Figure 3A–F), and the resulting images were analyzed using Imaris software to determine mitochondrial staining intensity, count, surface area, and volume. The cytokine cocktail had the largest effect on mitochondrial staining intensity, which is a measure of mitochondrial membrane potential, causing a significant decrease relative to DAPI (Figure 3G). TGF-β2, IL-6, and IL-1β treatments decreased mitochondrial complexity as quantified by the mitochondrial complexity index (MCI) (Figure 3H), which quantifies mitochondrial surface area relative to volume (see methods section). Mitochondria count was unchanged across treatment groups (Figure 3I); however, the cytokine cocktail significantly increased the average total mitochondrial volume, which may be due to mitochondrial swelling (Figure 3J). Overall, the cytokine cocktail had the most potent effects on mitochondrial dynamics, increasing overall volume, while decreasing the staining intensity.

NAD^+^ is a vital co-factor for key enzymes in both glycolysis and the TCA cycle. Studies in cancer models have associated an increased NADH/NAD^+^ ratio with increased flux through the TCA cycle [49]. NADP^+^ is another essential co-enzyme that, in its reduced form (NADPH), offers reducing equivalents for lipid and nucleotide biosynthesis and serves as a crucial antioxidant to maintain the pool of reduced glutathione. Motivated by the observed effects of cytokines on glycolysis and OXPHOS, we investigated their potential influence on the NADH/NAD^+^ and NADPH/NADP^+^ ratios. Differentiated ARPE-19 cells were treated with cytokines, and reduced and oxidized nicotinamide dinucleotides were quantified. Our findings revealed that neither the NADH/NAD^+^ ratio (Figure 3K), nor the NADPH/NADP^+^ ratio (Supplementary Figure S2) were altered significantly by cytokine treatments.

Though glycolysis and OXPHOS both contribute to ATP generation, OXPHOS is significantly more efficient, generating an estimated 30–36 molecules of ATP per glucose molecule, compared with just 2 ATP molecules derived from glycolysis [50]. Our analysis of lactate efflux and ECAR suggested that most cytokines elevated the rate of glycolysis. Conversely, the cytokines exhibited more varied effects on OXPHOS, as evidenced by the OCR. Thus, to decipher the net effects of these cytokines on ATP/ADP ratios in the RPE, we subjected differentiated ARPE-19 cells to cytokine treatment and assayed for ATP and ADP. Our results showed that while most cytokines prompted an increase in the ATP/ADP ratio, only the cytokine cocktail induced a significant increase.

### Effects of cytokines on lactate production are partly mediated through AKT

TNFα, TGF-β2, IL-6, and IL-1β activate signaling pathways known to regulate metabolism, including PI3K/AKT, MEK/ERK, and NF-κB [36,51–58]. We investigated the specific signaling pathways that may mediate the observed effects of cytokine treatments on metabolism in the RPE. For this, ARPE-19 cells were subjected to co-treatment with cytokines and inhibitors targeting the PI3K/AKT, MEK/ERK, and NF-κB pathways.

To assess the contribution of the PI3K/AKT and MEK/ERK pathway to cytokine-induced metabolic changes, cells were treated with cytokines, with or without the addition of the pan-AKT inhibitor, MK 2206, or the MEK inhibitor, PD 98059. Lactate concentrations were assayed in medium samples and western blot analysis on cell protein extracts was used to determine the presence of total AKT, phospho-AKT, ERK, and phospho-ERK.

**Figure 4:**
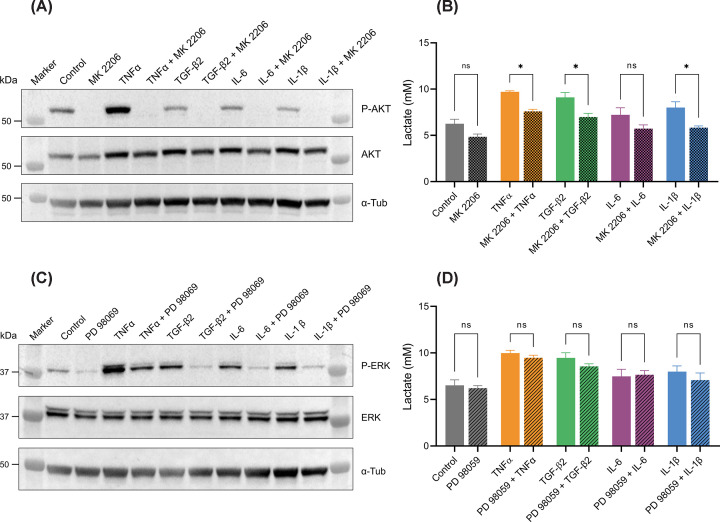
AKT mediates cytokine-induced metabolic effects (**A**) Western blot analysis of total and phospho-AKT in protein extracts from differentiated ARPE-19 cells following 24-h treatment with 20 ng/ml cytokines, administered with or without MK 2206. (**B**) Lactate assay performed on media samples from differentiated ARPE-19 cells following a 24-h treatment with 20 ng/ml cytokines, with or without MK 2206 (*n* = 3 independent experiments). (**C**) Western blot analysis of total and phospho-ERK in protein extracts from differentiated ARPE-19 cells following a 24-h treatment with 20 ng/ml cytokines, administered with or without PD 98059. (**D**) Lactate assay performed on media samples from differentiated ARPE-19 following a 24-h treatment with 20 ng/ml cytokines, with or without PD 98059 (*n* = 3 independent experiments). Error bars show mean values ± SEM. Statistical significance is denoted as follows: *P<0.05, determined by one-way ANOVA with Šidák’s multiple comparison test.

Phospho-AKT was detected in all samples not treated with MK 2206 (Figure 4A), suggesting this pathway is active under basal conditions. Among the cytokines, only TNFα significantly increased phospho-AKT abundance, indicating its role in promoting activation of this pathway. As expected, phospho-AKT was undetectable in samples treated with MK 2206, validating the inhibitor’s efficacy. All cytokines increased the level of phospho-ERK relative to total ERK, with TNFα exhibiting the most potent effect (Figure 4C). Phospho-ERK was largely eliminated with PD 98059 treatment, indicating the efficacy of the inhibitor. The increase in lactate production induced by TNFα, TGF-β2, and IL-1β was partially suppressed by AKT inhibition. Conversely, PD 98059 treatment did not significantly influence cytokine-induced lactate production in either the cytokine-treated or control groups (Figure 4D).

Nuclear factor kappa B (NF-κB) is a protein complex that regulates gene transcription in response infection and inflammation. It is the primary target of TNFα signaling, although it has been reported to be a downstream target for a variety of cytokines [40,58,59]. To investigate the effects of TNFα, TGFβ2, IL-6, and IL-1β on NF-κB activation, we adapted a dual fluorescence reporter system in-house [60]. In this modified system, eGFP is constitutively expressed under the control of a CMV promoter, while expression of nucTomato red fluorescent protein is regulated by a 5X NF-κB response element (NRE) (Supplementary Figure S3A). Consequently, NF-κB activation increases in the Tomato:GFP ratio in response to treatment. The reporter system was stably integrated into ARPE-19 cells using lentiviral transduction. Treatment with TNFα led to a sizeable induction of the reporter system (Supplementary Figure S3B), while TGFβ2, IL-6, and IL-1β triggered a negligible response in NF-κB activity. Based on these observations, we focused on investigating NF-κB as a potential mediator of TNFα’s metabolic effects.

**Figure 5:**
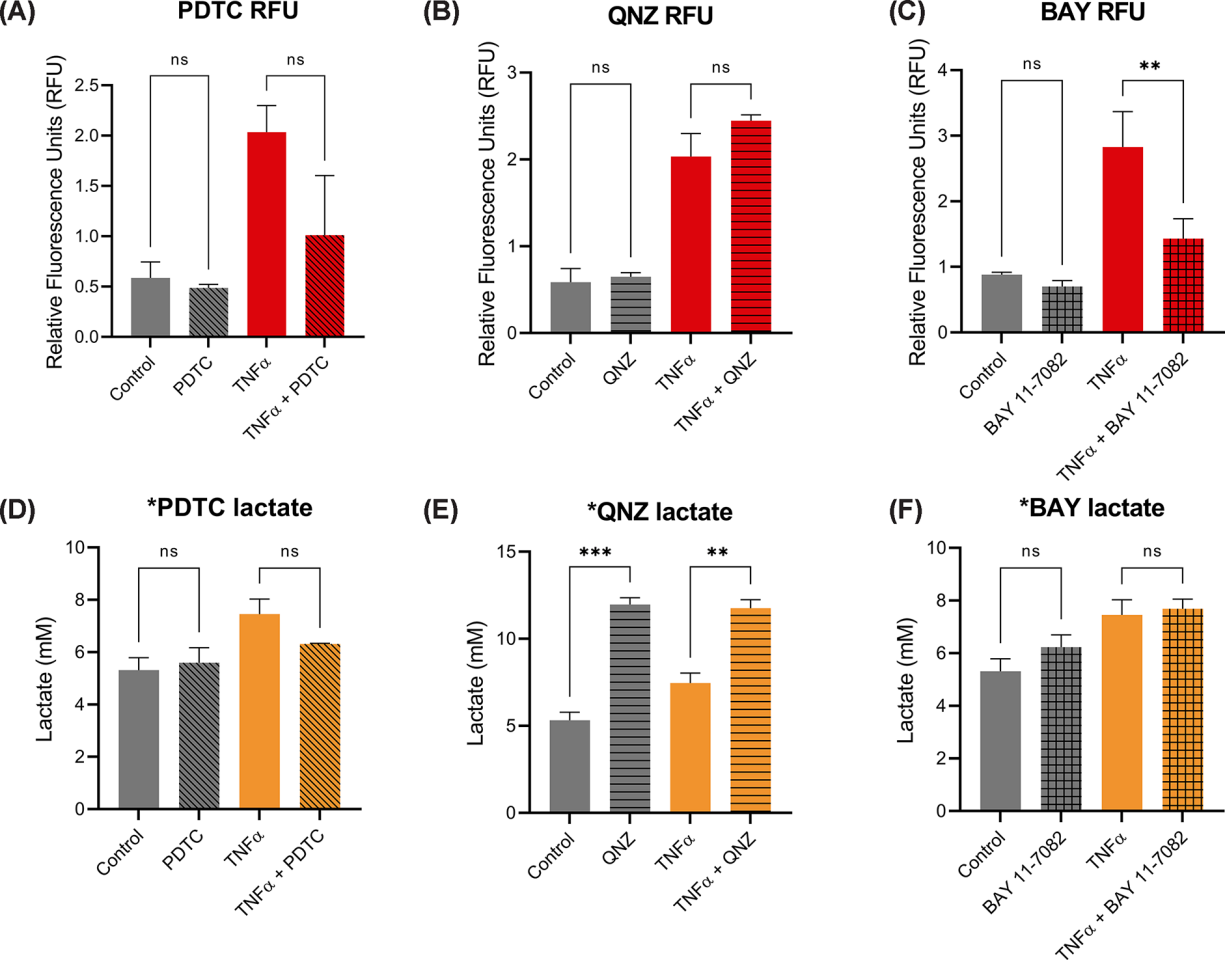
TNFα metabolic effects are not mediated by NF-κB Tomato:GFP ratios in 5XNRE ARPE-19 cells were measured following 24-h treatment with 20 ng/ml TNFα, with and without (**A**) 100 μM PDTC, (**B**) 20 nM QNZ, and (**C**) 5 μM BAY 11-7082. Lactate assay of media samples from 5XNRE ARPE-19 cells following 24-h treatment with 20 ng/ml TNFα, with and without (**D**) 100 μM PDTC, (**E**) 20 nM QNZ, and (**F**) 5 μM BAY 11-7082; *n* = 3 independent experiments. Error bars show mean values ± SEM. Statistical significance: *P<0.05, **P<0.01, ***P<0.001, determined by one-way ANOVA with Dunnett’s multiple comparison test.

The 5XNRE ARPE-19 cell line was used to validate three NF-κB inhibitors: PDTC, QNZ, and BAY 11-7082. Cells were cultured for 6 weeks to maximize differentiation, then treated with 20 ng/ml TNFα with or without each inhibitor for 24 h, then imaged to quantify fluorescence, while medium samples were assayed for lactate. In all of these experiments, TNFα stimulated the NRE reporter and increased lactate production (Figure 5). PDTC and BAY 11-7082 both inhibited TNFα-induced reporter induction (Figure 5A,C). There was considerable variation between experiments using PDTC and treatment-associated changes were not statistically significantly different from control (Figure 5A). Surprisingly, QNZ did not inhibit TNFα-induced reporter activity in these cells (Figure 5B). PDTC resulted in apparent inhibition of TNFα-induced lactate production, but the effect was not statistically significant (Figure 5D). Unexpectedly, treatment with QNZ led to a marked increase in lactate production in the presence or absence of TNFα (Figure 5E). Lastly, BAY 11-7082, the most effective inhibitor in these cells of the TNFα-induced NF-κB reporter, did not prevent TNFα-induced lactate production (Figure 5F). These data demonstrate that NF-κB signaling is not the major mediator of TNFα-induced lactate production in ARPE-19 cells.

### Cytokines influence expression of glycolytic genes

To further examine the mechanisms underlying the observed cytokine-induced changes in glycolysis, we investigated whether there were concomitant alterations in the expression of genes encoding key glycolytic enzymes. ARPE-19 cells were treated with cytokines for 24 h, and RT-qPCR of RNA extract was performed to determine relative expression of glycolytic enzymes LDHA, LDHB, HK2, PKM2, and the glucose transporters GLUT1 and GLUT3.

**Figure 6:**
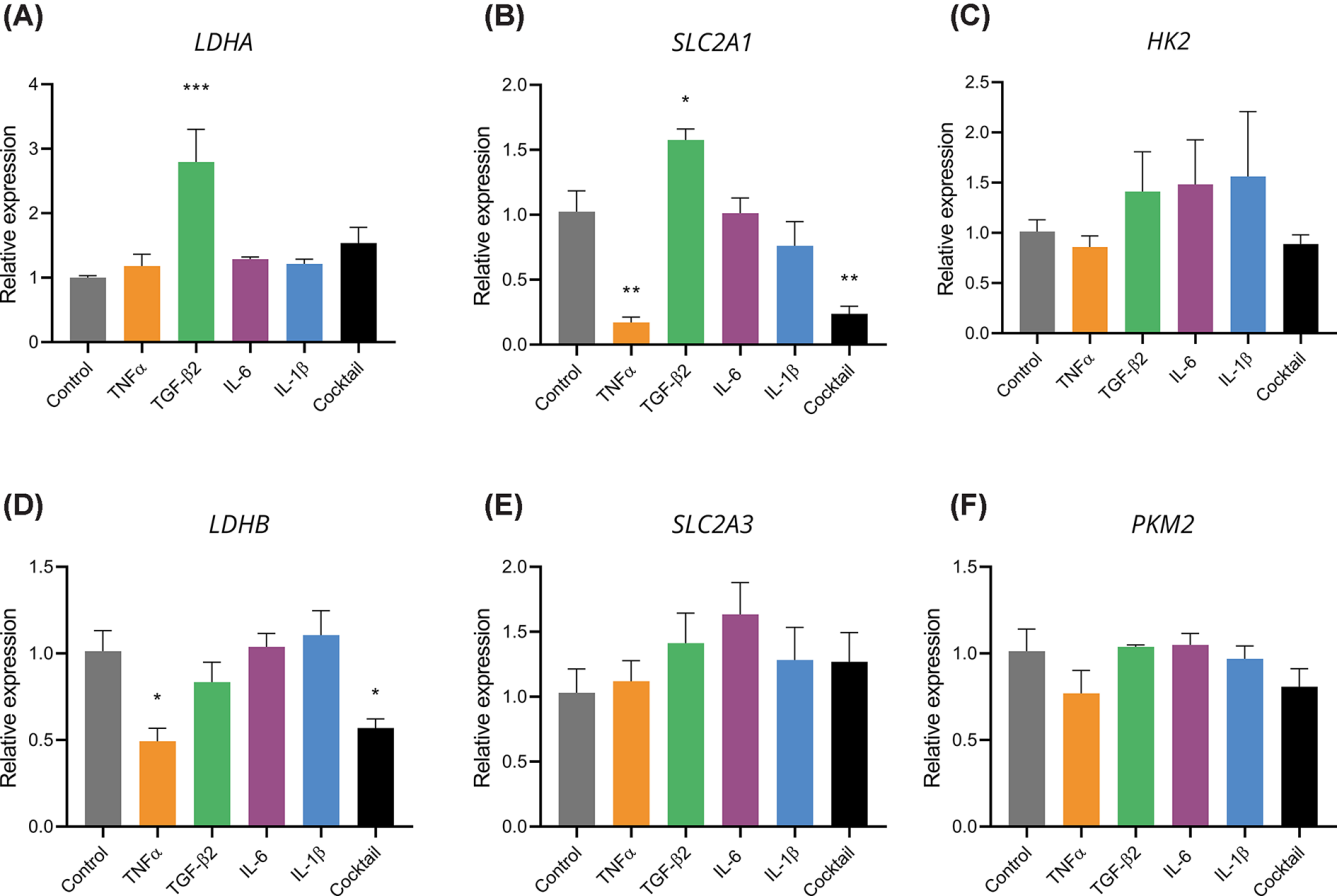
Cytokines alter expression of GLUT-1 and LDH isoforms Differentiated ARPE-19 cells were treated with 20 ng/ml cytokines for 24 h, followed by RNA extraction and RT-qPCR analysis. Expression levels of (**A**) LDHA, (**B**) SLC2A1, (**C**) HK2, (**D**) LDHB, (**E**) SLC2A3, and (**F**) PKM2 were normalized to the housekeeping gene HPRT1 and compared with vehicle-treated controls. *n* = 3 independent experiments. Error bars show mean ± SEM. Statistical significance: *P<0.05, **P<0.01, ***P<0.001, determined by one-way ANOVA with Dunnett’s multiple comparison test.

Most of the individual cytokines and the cocktail did not significantly increase the expression of any of the target genes. However, TGF-β2 significantly up-regulated the expression of both *LDHA* and *SLC2A1* (Figure 6A,B). These elevations in gene expression align with the increased glucose consumption and lactate production observed upon TGF-β2 treatment. Moreover, there was a decrease in the expression of *LDHB* upon treatment with TNFα and the cytokine cocktail (Figure 6D). Considering the role of the LDHB subunit in promoting the conversion of lactate to pyruvate, a reduction in its expression relative to LDHA could tilt the balance toward the conversion of pyruvate to lactate. This hypothesis is supported by the lactate assay and ECAR data, which demonstrate an increase in lactate efflux upon treatment with TNFα and the cytokine cocktail.

Both TNFα and the cytokine cocktail unexpectedly caused a sizeable decrease in the expression of *SLC2A1* (Figure 6B), a finding seemingly at odds with the increased glucose consumption observed with TNFα and cytokine cocktail treatment (Figure 1A,B). In contrast, none of the cytokines caused a statistically significant change in *SLC2A3* expression (Figure 6E), so *SLC2A3* is not compensating for the decrease in *SLC2A1* expression. There were no significant shifts in the relative expression of *HK2* or *PKM2* with cytokine treatment (Figure 5C,F). However, there was a high degree of variability in *HK2* expression, likely due to low basal expression of this gene in the cells. These results suggest a complex and multifaceted regulation of glycolytic metabolism by cytokines at the level of gene expression.

## Discussion

During aging and in age-related diseases such as AMD, the cytokines TNFα, TGF-β2, IL-6 and IL-1β rise in abundance within the RPE/choroid [20], imposing a chronic inflammatory burden. This perpetual inflammation by cytokines provokes numerous pernicious effects, including tissue damage, oxidative stress, fibrosis, and necrosis [61–64]. Our study shows that these inflammatory cytokines perturb RPE metabolism, potentially destabilizing the retina’s intricate metabolic balance.

Of the four cytokines investigated, TNFα had the most potent and consistent metabolic effects on the RPE. Exposure to TNFα significantly increased lactate production and glucose consumption in ARPE-19 cells and rat eyecups, and in primary RPE cells isolated from two of three human eye donors. Each of the individual cytokines elicited variable responses among the primary RPE isolates. While the cause of this variability is unclear, it may reflect genetic and epigenetic variations among the donors.

In ARPE-19 cells, TNFα induced the largest increase in both basal and maximal ECAR, supporting a recent study that reported increased glycolytic capacity in fibroblast-like synoviocytes following TNFα treatment [65]. On the other hand, this result contradicts the findings of another study that showed a decrease in both basal and maximal ECAR in human fetal RPE (hfRPE) cells following TNFα treatment [66]. This discrepancy may be due to hfRPE-specific responses or could be related to the longer treatment duration (5 days versus 24 h in our study) leading to different, less direct metabolic outcomes. Increased glycolytic reserve capacity can be explained by changes in glycolytic gene expression. For example, while TNFα does not increase *LDHA* expression, it does suppress expression of *LDHB*, elevating the LDHA/LDHB ratio and promoting the conversion of pyruvate to lactate. Interestingly, TNFα treatment decreased *SLC2A1* expression, which was unexpected given that the treatment triggers an increase in glucose uptake, and it contrasts with other studies that have reported increases in *SLC2A1* expression following TNFα treatment in different cell types [23,65].

We observed substantial increases in both phospho-AKT and phospho-ERK with TNFα treatment, in line with the activation of both the PI3K/AKT [67] and the MEK/ERK [68,69] pathways in other cell types. Our experiments showed that inhibiting AKT partially mitigated the lactate production induced by TNFα treatment. AKT is a kinase with several downstream targets that could influence metabolism, including glycogen synthase kinase 3 (GSK3) and the mammalian target of rapamycin complex 1 (mTORC1) [70]. Moreover, AKT can directly phosphorylate glycolytic enzymes, activating 6-phosphofructo-2-kinase (PFK2) [71] and increasing association of HK2 with mitochondria [72]. Further research is required to understand these mechanisms more clearly.

Similar to TNFα, TGF-β2 potently increases both lactate production and glucose consumption in ARPE-19 cells, as well as increases glucose consumption in rat eyecups and in two of three primary human RPE isolates. The consistent glucose uptake across models may be due to the fact that TGF-β2 was the sole cytokine to significantly increase expression of *SLC2A1*, the gene coding for the glucose transporter GLUT1.

The absence of a detectable increase in lactate efflux in rat eyecups and primary human RPE cells suggests that TGF-β2 promotes glucose consumption, but the carbons are channeled into metabolites other than lactate. These carbons are unlikely to be processed in the TCA cycle, as no increase in OCR was observed following TGF-β2 treatment. TGF-β2 may promote flux through the pentose phosphate pathway (PPP) and increase glucose carbon incorporation into ribose, which may explain this discrepancy [73]. However, this phenomenon would typically be associated with an increase in the NADPH/NADP^+^ ratio [74], which we did not observe in our study. TGF-β is a well-established key driver of epithelial-to-mesenchymal transition (EMT) in various tissues [75]. Notably, EMT in the RPE, driven by factors such as TGF-β, is thought to contribute to the development of eye diseases such as AMD and proliferative vitreoretinopathy [76,77]. Therefore, induction of EMT by TGF-β may promote the incorporation of glucose carbons into extracellular matrix (ECM) and cytoskeletal proteins, as well as other macromolecules associated with EMT [78] and reduce the amount of glucose available to the photoreceptors in the outer retina.

AKT inhibition significantly mitigated TGF-β2-induced lactate production; however, this occurred despite TGF-β2 failing to significantly elevate phospho-AKT levels relative to total AKT. Moreover, TGF-β2 has previously been reported to stimulate type I collagen expression through PI3K/AKT in ARPE-19 cells, resulting in increased phospho-PI3K and AKT activity [79]. Given that TGF-β2 failed to increase phospho-AKT levels in our study, and that MK 2206 also suppressed lactate production in the control cells, it is unclear whether the metabolic effects are mediated by the PI3K/AKT pathway. As seen with TGF-β treatment in other cell types [26,38], significant increases in the expression of *LDHA* and *SLC2A1* were induced by TGF-β2, in line with the heightened lactate production and glucose consumption observed in ARPE-19s.

Among the cytokines investigated in this study, IL-6 had the most limited impact on RPE lactate production and glucose consumption across all three models. IL-6 did not affect glycolytic gene expression in ARPE-19 cells, and elicited only minor effects on ECAR and OCR. The limited metabolic influence of IL-6 on the RPE is surprising given that IL-6 treatment has been shown to promote both glucose uptake and lactate efflux in skeletal muscle cells [29], adipocytes [28], and colorectal cancer cells [30]. Moreover, both components of the IL-6 receptor complex (IL-6R), namely IL-6Rα and gp130, are expressed in cultured RPE cells [45]. The observed increase in phospho-ERK relative to total ERK suggests that downstream signaling pathways are being activated be IL-6 treatment. However, it appears that IL-6 may lack sufficient downstream metabolic targets to significantly alter RPE metabolism.

IL-1β significantly elevated lactate production in ARPE-19 cells and one primary RPE donor, while having no effect on rat eyecups. Moreover, IL-1β elicited a significant rise in basal respiration, indicating a subtle shift toward ATP generation via OXPHOS rather than glycolysis. This stands in contrast with other cytokines investigated, which generally favored ATP production via glycolysis. Despite the rise in basal respiration, no increase was observed in mitochondrial count or volume; instead, it coincided with decreased mitochondria staining intensity. As with TGF-β2, IL-1β failed to increase phospho-AKT levels, making it unlikely that the PI3K/AKT pathway mediates the metabolic effects of IL-1β, despite MK 2206 significantly nullifying IL-1β-induced lactate production.

The cytokine cocktail was used to mimic the *in vivo* setting of an inflamed RPE and choroid more closely, where multiple cytokines simultaneously activate intracellular signaling. In support of this, the cocktail was the most consistent in eliciting metabolic responses across different experimental models. It was the only treatment that increased lactate production and glucose consumption in ARPE-19 cells, rat eyecups, and all three primary RPE donors. Moreover, the cocktail amplified lactate production in ARPE-19 cells more than any two-cytokine combination. The cocktail also significantly elevated the maximal ECAR, indicating an increased glycolytic reserve capacity of the cells.

The cytokine cocktail elicited similar effects on gene expression to TNFα, decreasing both *LDHB* and *SLC2A1* expression. However, the most striking metabolic effects were observed in respiration: the cocktail increased ATP production through both glycolysis and OXPHOS under basal conditions, and significantly increasing the ATP/ADP ratio. This was unexpected, considering the only other cytokine to significantly increase basal respiration was IL-1β which had a relatively minor effect.

Upon treatment with the mitochondrial uncoupler FCCP, cytokine cocktail treatment doubled maximal respiration, accompanied by a significant increase in total mitochondrial volume per cell and a decrease in mitochondria staining intensity. Given the sizable increases in oxygen consumption, the decreased staining intensity may reflect mitochondrial uncoupling within the RPE, rather than decreased OXPHOS. MitoTracker Red CMXRos accumulation in mitochondria is dependent on mitochondrial membrane potential so treatment with FCCP decreases the staining intensity of this fluorescent dye [80]. Moreover, recent reports suggest that succinate oxidation can drive O_2_ consumption decoupled from ATP synthesis in the RPE [7]. In addition, various cytokines have been implicated in promoting mitochondrial uncoupling. For example, TNFα induces mitochondrial uncoupling in primary murine hepatocytes [81] and isolated rat mitochondria [82], while overexpression of IL-6 in rats induces uncoupling protein 1 (UCP1) in brown adipose tissue [83]. IL-1β also promotes mitochondrial depolarization in adipocytes [84]. Thus, the cytokine cocktail may induce mitochondrial uncoupling in the RPE, forcing cells to increase mitochondrial volume to compensate for less efficient ATP production, leading to sizeable increases in O_2_ consumption.

Herein, we demonstrate that cytokines not only alter glucose metabolism in the RPE but exhibit effects that are unique to each of the prevalent cytokines found within this tissue. Importantly, when combined in a manner that better reflects the chronic inflammatory environment of the RPE and choroid during aging and disease, these cytokines operate synergistically to alter glucose metabolism, ultimately promoting both glycolysis and O_2_ consumption. Given the position of the RPE between the choroid and neural retina, augmented glucose and O_2_ consumption by the RPE could restrict the availability of these nutrients for photoreceptor function, ultimately compromising vision. Moreover, given the complex exchange of metabolites between the RPE and photoreceptors, cytokine-driven metabolic dysregulation in the RPE may upset the outer-retinal metabolic ecosystem. Given the abundance of cytokines in this tissue during aging and in age-related diseases, our study suggests potential therapeutic opportunities by targeting cytokines or their associated signaling pathways within the RPE. However, further investigations are warranted to more fully understand the functional consequences that cytokine-induced changes in RPE metabolism have on metabolite exchange and retinal function.

## Methods

### Cell culture

The human retinal pigment epithelial cell line ARPE-19 was sourced from the American Type Cell Collection (Manassas, VA). ARPE-19 cells were seeded at a density of 5 × 10^4^ cells/cm^2^ and cultured for 6–8 weeks in a 50/50 mix of Dulbecco’s modified Eagle’s medium (DMEM) (11996-065, Thermo Fisher Scientific-Gibco, Grand Island, NY) and F12 (11765-065, Thermo Fisher Scientific-Gibco, Grand Island, NY) supplemented with 10% fetal calf serum (FCS) (Merck-Sigma Aldrich, St Louis, MO), 1% Glutamax (Thermo Fisher Scientific-Gibco, Grand Island, NY), 100 U/ml penicillin/streptomycin (Thermo Fisher Scientific-Gibco, Grand Island, NY) and 10 mM nicotinamide (Merck-Sigma Aldrich, St Louis, MO) to promote differentiation as previously described [85]. Cells were maintained in a 37°C humidified incubator with 5% CO_2_ with the medium refreshed every 5 days.

### Isolation and culture of human retinal pigment epithelial cells

Retinal pigment epithelial cells were isolated from human cadaveric eyes as described previously [86]. The three donors were male, under the age of 50 with no history of eye disease. Briefly, the retinal pigment epithelium-choroid layer was dissected from the posterior eyecup and immersed in 0.25 mg/ml collagenase IA and 0.25 mg/ml collagenase IV (Merck-Sigma Aldrich, St Louis, MO) in phosphate buffered saline (PBS) with 2% fetal bovine serum (FBS) (Bovogen, Keilor East, Australia) for 30 min at 37°C and 5% CO_2_ in air. After the incubation, the epithelial cells were gently scraped off Bruch’s membrane as sheets under a dissecting microscope. The cell sheets were collected by density gradient centrifugation using 10% sucrose in PBS with 2% FBS, and subsequently plated in 50% Minimum Essential Medium Eagle alpha modification (MEM), 25% DMEM, and 25% F-12 medium, with 1X N1 Medium Supplement, 1X Non-Essential Amino Acids Solution, 1X GlutaMAX Supplement, 0.25 mg/ml taurine, 0.02 μg/ml hydrocortisone, 0.013 ng/ml 3,3′,5-triiodo-L-thyronine, 100 U/ml Penicillin-100 μg/ml Streptomycin (all obtained from Merck-Sigma Aldrich or Thermo Fisher Scientific-Gibco, Grand Island, NY), and 2% FBS. Over 2 to 3 weeks in culture, the cells achieved confluent cobblestone morphology, and were stored frozen in liquid nitrogen. Cell phenotype was verified by indirect immunocytofluorescence labelling for expression of retinal pigment epithelial cell markers (i.e. cytokeratin 8, retinal pigment epithelium-specific 65 kDa protein, and zonula occludens 1) and absence of α-smooth muscle actin.

### Eyecup dissection and treatment

All experiments using rat eyes were performed at the University of Adelaide. Eyes were scavenged from Sprague-Dawley rats that were killed using CO_2_ asphyxiation. Whole eyes were immediately enucleated and immersed in ice cold Hank’s balanced salt solution (HBSS) (Thermo Fisher Scientific-Gibco, Grand Island, NY) supplemented with 100 U/ml penicillin/streptomycin. Excess connective tissue was carefully removed from the sclera, and a minor incision, located 1–2 mm anterior to the cornea, was made using a scalpel. The cornea, lens and vitreous were then dissected to expose the posterior eyecup. After carefully detaching the retina, the remaining eyecup was immediately transferred to a petri dish with DMEM containing 5.5 mM glucose and 100 U/ml penicillin/streptomycin. Each eyecup was then incubated in an individual well of a 12-well tissue culture plate (Corning, NY) containing 5.5 mM glucose and 100 U/ml penicillin/streptomycin with or without cytokines for 24 h.

### Lactate and glucose assay

Following treatment, 100 μl samples were taken from the cell culture medium and immediately frozen at −80°C for subsequent analysis. The lactate assay was performed using 1 μl of each sample with 9 μl of water in a 96-well cell culture plate, alongside lactate standards for comparison. Approximately 190 μl of reaction mixture containing 2.5 U/L lactate oxidase (Merck-Sigma Aldrich, St Louis, MO), 50 U/L horseradish peroxidase (Merck-Sigma Aldrich, St Louis, MO), and 20 μM Amplex Red (Thermo Fisher Scientific-Invitrogen, Grand Island, NY) in 0.1 M sodium phosphate buffer (pH 7.5) was added to each well. The reactions were protected from light and incubated for 30 min at 37°C. Fluorescence was measured (*E*x 520 nm, *E*m 580–640 nm) using a GloMax Discover system (Promega, Madison, WI). Glucose assays were performed using a similar method, however with a reaction mixture containing 135 U/L glucose oxidase (Merck-Sigma Aldrich, St Louis, MO), 50 U/L horseradish peroxidase (Merck-Sigma Aldrich, St Louis, MO), and 20 μM Amplex Red (Thermo Fisher Scientific-Invitrogen, Grand Island, NY) in 0.1 M sodium phosphate buffer (pH 6).

### Cytokine and inhibitor treatment

Cells were pre-equilibrated in DMEM containing 5.5 mM glucose for 24 hours prior to the experiment. For cytokine treatments, ARPE-19 cells were treated for 24 hours with 20 ng/mL TNFα (Merck-Sigma Aldrich, St Louis, MO), TGF-β2 (Merck-Sigma Aldrich, St Louis, MO), IL-6 (Thermo Fisher Scientific-Gibco, Grand Island, NY), IL-1β (Merck-Sigma Aldrich, St Louis, MO), or a cytokine cocktail containing all four cytokines. For AKT and MEK inhibition, ARPE-19 cells were treated with 10 μM MK 2206 (Cayman Chemical, Ann Arbor, MI) or 30 μM PD 98059 (Cayman Chemical, Ann Arbor, MI), respectively. For NF-κB inhibition studies, 5XNRE ARPE-19 cells were treated with 100 μM PDTC (Cayman Chemical, Ann Arbor, MI), 20 nM QNZ (Cayman Chemical, Ann Arbor, MI), or 5 μM BAY 11-7082 (Cayman Chemical, Ann Arbor, MI).

### Mitochondria imaging

ARPE-19 cells were seeded on 0.1% gelatin-coated class coverslips and cultured for 6 weeks to differentiate. Cells were pre-equilibrated in 5.5 mM DMEM, then treated with 20 ng/ml cytokines in 5.5 mM DMEM for 24 h. Medium was then replaced with fresh medium containing 250 nM MitoTracker Red CMXRos (Thermo Fisher Scientific-Invitrogen, Grand Island, NY), and cells were incubated at 37°C for 45 min. After washing with PBS, cells were fixed with 4% paraformaldehyde for 15 min at 37°C, washed thrice with PBS, and mounted onto glass slides using ProLong Gold antifade reagent with DAPI (Thermo Fisher Scientific-Molecular Probes, Grant Island, NY). Images were acquired on an LSM980 Airyscan microscope (ZEISS) and processed using Imaris v9.9.1 (Bitplane) for mitochondrial surface rendering. The mitochondrial complexity index (MCI) was calculated from the formula provided by Vincent et al. [87].

$$MCI=\frac{{SA}^{3}}{16\pi^{2}V^{2}}$$

Where MCI = mitochondrial complexity index, SA = mitochondrial surface area, and V = mitochondrial volume.

### Seahorse extracellular flux analysis

Real time extracellular acidification rate (ECAR) and oxygen consumption rate (OCR) were measured using the Seahorse XFe96 Extracellular Flux Analyzer (Agilent Technologies, Santa Clara, CA). ARPE-19 cells were seeded on Seahorse XF96 V3 PS cell culture microplates pre-coated with 0.2% gelatin in PBS and cultured for 6 weeks in growth medium to achieve maximal differentiation. Cells were pre-equilibrated for 24 h in DMEM with 5.5 mM glucose, supplemented with 10% FCS (Merck-Sigma Aldrich, St Louis, MO), and 1% Glutamax (Thermo Fisher Scientific-Gibco, Grand Island, NY), then 20 ng/ml cytokines were added for 24 h. For the Mito Stress Test, cells were incubated for 1 h in a 37°C, 5% CO_2_ humidified incubator with assay medium composed of Seahorse XF Base Medium (Agilent Technologies, Santa Clara, CA) without Phenol Red, containing 1 mM pyruvate, 2 mM L-glutamine, and 10 mM glucose, at pH 7.4. Drugs were added at 16, 36, and 56 min with final concentrations of 2.5 μM oligomycin, 0.5 μM FCCP, and 1 μM rotenone/antimycin A, respectively. For the Glycolytic Stress Test, cells were incubated in similar environment, but the assay medium contained 2 mM L-Glutamine alone added to Seahorse XF Base Medium. Drugs injections were administered at the same intervals as the Mito Stress Test, but with final concentrations of 10 mM glucose, 2.5 μM oligomycin, and 50 mM 2-deoxyglucose.

### Reduced and oxidized nicotinamide dinucleotide (NAD) and nicotinamide dinucleotide phosphate (NADP) extraction and assay

After cytokine treatment, the medium was removed from the ARPE-19 cells and replaced with 100 μl of either 0.2 M HCl (for NAD^+^/NADP^+^ extraction) or 0.2 M NaOH (for NADH/NADPH extraction) per well in a 6-well tissue culture plate. Cells were scraped, transferred into 1.5 ml microfuge tubes, incubated at 50°C for 10 min, then cooled on ice for 5 min. To neutralize the samples, 100 μl of either 0.1 M NaOH (for NAD^+^/NADP^+^ extracts) or 0.1 M HCl (for NADH/NADPH extracts) was added dropwise while vortexing. After sonication for 15 min in a bath sonicator, the samples were centrifuged 20,000 × ***g*** for 5 min at 4°C. The supernatants were then transferred to new ice-cold microfuge tubes and protected from light.

The NAD^+^/NADH and NADP^+^/NADPH levels were determined using a cycling assay, modified from previously reported methods [88]. Approximately 5 μl of 2, 1, 0.5, 0.1, 0.05 and 0 μM NAD^+^ and NADP^+^ standards were added to a 96-well tissue culture plate. NAD^+^/NADP^+^ and NADH/NADPH extracts were diluted 1/5 with MQ water, and 5 μl of these diluted extracts were added to per well. For the NAD^+^/NADH assay, 95 μl of assay mixture containing 50 μg/ml alcohol dehydrogenase (Merck-Sigma Aldrich, St Louis, MO), 10% EtOH, 3 mM PMS (Cayman Chemical, Ann Arbor, MI), 0.5 mM MTT (Cayman Chemical, Ann Arbor, MI), and 4 mM EDTA in 0.1 M bicine buffer pH 8 was added to each well. For the NADP^+^/NADPH assay, 95 μl of assay mixture containing 1.5 U/ml glucose-6-phosphate dehydrogenase (Merck-Sigma Aldrich, St Louis, MO), 1 mM glucose-6-phosphate, 3 mM PMS (Cayman Chemical, Ann Arbor, MI), 0.5 mM MTT (Cayman Chemical, Ann Arbor, MI), and 4 mM EDTA in 0.1 M bicine buffer pH 8 was added to each well. Absorbance at 560 nm was measured every minute for 30 min using a Glomax plate reader (Promega, Madison, WI). To normalize the NAD^+^/NADP^+^ and NADH/NADPH values, the protein concentration was determined using a Pierce bicinchoninic acid (BCA) assay (Thermo Fisher Scientific, Grant Island, NY).

### ATP/ADP extraction and assay

Following cytokine treatment, the medium was removed from ARPE-19 cells and replaced with 500 μl of ice-cold extraction buffer (containing 10 mM Tris-HCl at pH 7.5, 1 mM EDTA, and 0.05% digitonin) per well in a 6-well tissue culture plate. Cells were scraped and pipetted into cold microfuge tubes, incubated on ice for 15 min to allow for cell lysis, centrifuged at 15,000 × ***g*** for 10 min at 4°C, and the supernatant transferred to ice-cold microfuge tubes.

ATP and ADP were determined using a firefly luciferase-based assay. An ADP-to-ATP conversion mix was prepared with 2.5 U/ml pyruvate kinase (Merck-Sigma Aldrich, St Louis, MO) and 2 mM phosphoenolpyruvate (PEP) in ATP assay buffer (containing 0.1 M tris-acetate buffer at pH 7.5, 0.1 M MgCl_2_, 1 mM DTT, and 0.1% BSA). Approximately 50 μl of the ATP/ADP extract was combined with 50 μl of the conversion mix and incubated at room temperature for 10 min, then chilled on ice to halt the reaction. For the non-converted (ATP) samples, 50 μl of the extract was mixed with 50 μl of ATP assay buffer to ensure equivalent dilution of converted samples. The ATP assay mix was prepared by adding 5 μg/ml firefly luciferase and 100 μM luciferin to the ATP assay buffer. Approximately 50 μl of ATP standards (0, 2, 4, 6, 8, and 10 μM concentrations), along with converted and unconverted samples, were added to an opaque white OptiPlate-96 (Perkin Elmer, Waltham, MA). The Glomax (Promega, Madison, WI) plate reader was programmed to inject 50 μl of assay mix per well, with an integration time of 10 s. ADP concentrations were determined by subtracting the unconverted ATP value from the ADP-to-ATP conversion value for each extract.

### Western blot

Following cytokine treatment, ARPE-19 cells in 6-well plates were washed once with ice cold 1× PBS, lysed in 100 μl ice-cold RIPA buffer (150 mM NaCl, 1% IGEPAL, 0.5% sodium deoxycholate, 0.1% SDS, 1 mM DTT, 1 mM PMSF, 1× PI cocktail (Merck-Sigma Aldrich, St Louis, MO), and 1× PhosSTOP (Roche, Switzerland) in 50 mM Tris, pH 8), scraped off the plate and transferred to ice-cold microfuge tubes. The lysate was agitated on a rotating platform at 4°C for 30 min, centrifuged at 14,000 RPM for 30 min, supernatants transferred to new tubes on ice and protein concentration quantified using a Pierce BCA kit (Thermo Fisher Scientific, Grant Island, NY).

For Western blotting, 40 μg of protein was separated on a 12% Mini-PROTEAN TGX Precast PAGE gel (Bio-Rad, Hercules, CA) and transferred onto a nitrocellulose membrane using the Trans-blot Turbo transfer system (Bio-Rad, Hercules, CA). The membranes were blocked with 5% skim milk in 1× PBST for 1 h at room temperature, then incubated overnight at 4°C with primary antibodies (all diluted 1:1000 in 2% skim milk in PBST), including rabbit anti-total ERK1/2 (4695S, Cell Signaling Technology, Danvers, MA), rabbit anti-phospho-ERK1/2 (4370S, Cell Signaling Technology, Danvers, MA), rabbit anti-total AKT (9272S, Cell Signaling Technology, Danvers, MA), rabbit anti-phospho-AKT (S473) (4060S, Cell Signaling Technology, Danvers, MA), or rat anti-α-tubulin antibody (MA1-80017, Thermo Fisher Scientific-Invitrogen, Grand Island, NY). Following three washes with 1× PBST, the membranes were incubated with HRP-conjugated secondary antibodies (diluted in 2% skim milk in 1× PBST) for 1 h at room temperature on a rocking platform. The secondary antibodies used were goat anti-rabbit IgG (1:10,000, Pierce and Life Technologies, Australia) and anti-rat IgG (1:10,000, Abcam, Cambridge, MA). Membranes were developed using Clarity Western ECL Substrate (1705061, Bio-Rad, Hercules, CA) and imaged using a ChemiDoc imaging system (Bio-Rad, Hercules, CA).

### 5XNRE reporter assay

A NF-κB reporter system was generated based on the dual fluorescence reporter system developed in-house [60]. The 5XNRE sequences were sourced from the pSIRV-NF-κB-eGFP plasmid (Addgene Plasmid #118093) and cloned into our dual fluorescence reporter construct, upstream of the nucTomato gene. Lentiviral particles for transduction were prepared by transiently transfecting HEK 293T cells with the pLV 5XNRE expression vector, co-transfected with the packaging plasmid, psPAX2, and the envelope plasmid, pMD2.G. The lentiviral particles were harvested from the medium of the HEK 293T cells 72 h post-transfection and then used to transduce ARPE-19 cells for 48 h, which were selected over 2–3 passages using 140 μg/ml hygromycin (Merck-Sigma Aldrich, St Louis, MO). For NF-κB inhibition studies, 5XNRE ARPE-19 cells were cultured for 6 weeks for maximal differentiation, then treated with 20 ng/ml cytokines in DMEM containing 5.5 mM glucose, with or without NF-κB inhibitors, for 24 h. Following the treatment, cells were imaged for green and red fluorescence using a ZOE Fluorescent Cell Imager (Bio-Rad, Hercules, CA), and the resulting fluorescence was quantified with ImageJ software v1.53t.

### Quantitative reverse transcription PCR (qRT-PCR)

Total RNA was extracted using the TRI Reagent (Thermo Fisher Scientific-Invitrogen, Grand Island, NY), and the concentration of the extracted RNA was determined using a NanoDrop 2000 spectrophotometer (Thermo Fisher Scientific). RNA was reverse transcribed into cDNA using M-MLV Reverse Transcriptase (Promega, Madison, WI). cDNA was then diluted 1/8, and amplification was performed the using FastStart Universal SYBR Green Master (ROX) (Roche, Switzerland) with 250 nM forward and reverse primers (Table 1) on the StepOnePlus real-time PCR system (Applied Biosystems). The qPCR protocol involved: 1 cycle of 95°C for 10 min, 45 cycles of 95°C for 15 s, and 60°C for 1 min. Melt curve analysis was performed to confirm amplification specificity. Gene expression levels were normalized to the reference gene, *HPRT1*, and fold changes in expression were calculated using the ΔΔCT method.

**Table 1:** Primers used for the amplification of glycolytic genes using qRT-PCR

Primer	Sequence
LDHA Forward	5′-AAGCTGTCATGGGTGGGTCC-3′
LDHA Reverse	5′-TCACCTCATAAGCACTCTCAACCA-3′
LDHB Forward	5′-GGGGGAACATGGCGACTCAA-3′
LDHB Reverse	5′-ACTTCATAGGCACTTTCAACCACC-3′
SLC2A1 Forward	5′-GATTGGCTCCTTCTCTGTGG-3′
SLC2A1 Reverse	5′-TCAAAGGACTTGCCCAGTTT-3′
SLC2A3 Forward	5′-AGCGATGGGGACACAGAAGGT-3′
SLC2A3 Reverse	5′-GGCATTTCCCTTGTCCGTCA-3′
HK2 Forward	5′-GCTGTTTGACCACATTGCCGA-3′
HK2 Reverse	5′-CGGATCAGAGCCACAACGTC-3′
PKM2 Forward	5′-CTGATTGCCCGTGAGGCAGA-3′
PKM2 Reverse	5′-GCCAGACTTGGTGAGGACGA-3′
HPRT1 Forward	5′-ACTTTGCTTTCCTTGGTCAGGC-3′
HRPT1 Reverse	5′-AGTCAAGGGCATATCCTACAACAAA-3′

## Supplementary Material

Supplementary Figures S1-S4

## Data Availability

All the data supporting the findings and conclusions of this study are available on Figshare (https://figshare.com/projects/Metabolic_Reprogramming_of_the_Retinal_Pigment_Epithelium_by_Cytokines_Associated_with_Age-related_Macular_Degeneration_/183850).

## Open Access

Open access for this article was enabled by the participation of University Of Adelaide in an all-inclusive *Read & Publish* agreement with Portland Press and the Biochemical Society under a transformative agreement with CAUL.

## Abbreviations

ADP: adenosine diphosphate
AMD: age-related macular degeneration
ATP: adenosine triphosphate
DAMP: damage-associated molecular pattern
2-DG: 2-deoxy-D-glucose
DMEM: Dulbecco’s modified Eagle’s medium
ECAR: extracellular acidification rate
eGFP: enhanced green fluorescent protein
EMT: epithelial-to-mesenchymal transition
ERK: extracellular signal-regulated kinase
FBS: fetal bovine serum
FCS: fetal calf serum
GLUT-1: glucose transporter 1
GSK3: glycogen synthase kinase 3
HK2: hexokinase 2
HPRT1: hypoxanthine phosphoribosyl transferase 1
IL-1β: interleukin 2β
IL-6: interleukin 6
JAK: Janus kinase
LDHA: lactate dehydrogenase A
LDHB: lactate dehydrogenase B
MEK: mitogen-activated protein kinase kinase
MEM: Minimum Essential Medium Eagle alpha modification
mTOR: mammalian target of rapamycin
NAD: nicotinamide adenine dinucleotide
NADP: nicotinamide adenine dinucleotide phosphate
NF-κB: nuclear factor κ-light-chain-enhancer of activated B cells
OCR: oxygen consumption rate
OXPHOS: oxidative phosphorylation
PBS: phosphate buffered saline
PFK2: phosphofructokinase 2
PI3K: phosphatidylinositol 3-kinase
PKM2: pyruvate kinase muscle isoform 2
PPP: pentose phosphate pathway
qRT-PCR: quantitative reverse transcription polymerase chain reaction
RPE: retinal pigment epithelium/epithelial
STAT3: signal transducer and activator of transcription 3
TCA: tricarboxylic cycle
TGF-β2: transforming growth factor β2
TNFα: tumor necrosis factor α
UCP1: uncoupling protein 1
