# Supplementary material for: Metabolic reprogramming of the retinal pigment epithelium by cytokines associated with age-related macular degeneration
Source: Biosci Rep. 2024 Apr 16;44(4):BSR20231904. doi: 10.1042/BSR20231904 (PMC11043024; doi:10.1042/BSR20231904)
Supplement: Supplementary Figures S1-S4 [file BSR-2023-1904_supp.pdf]

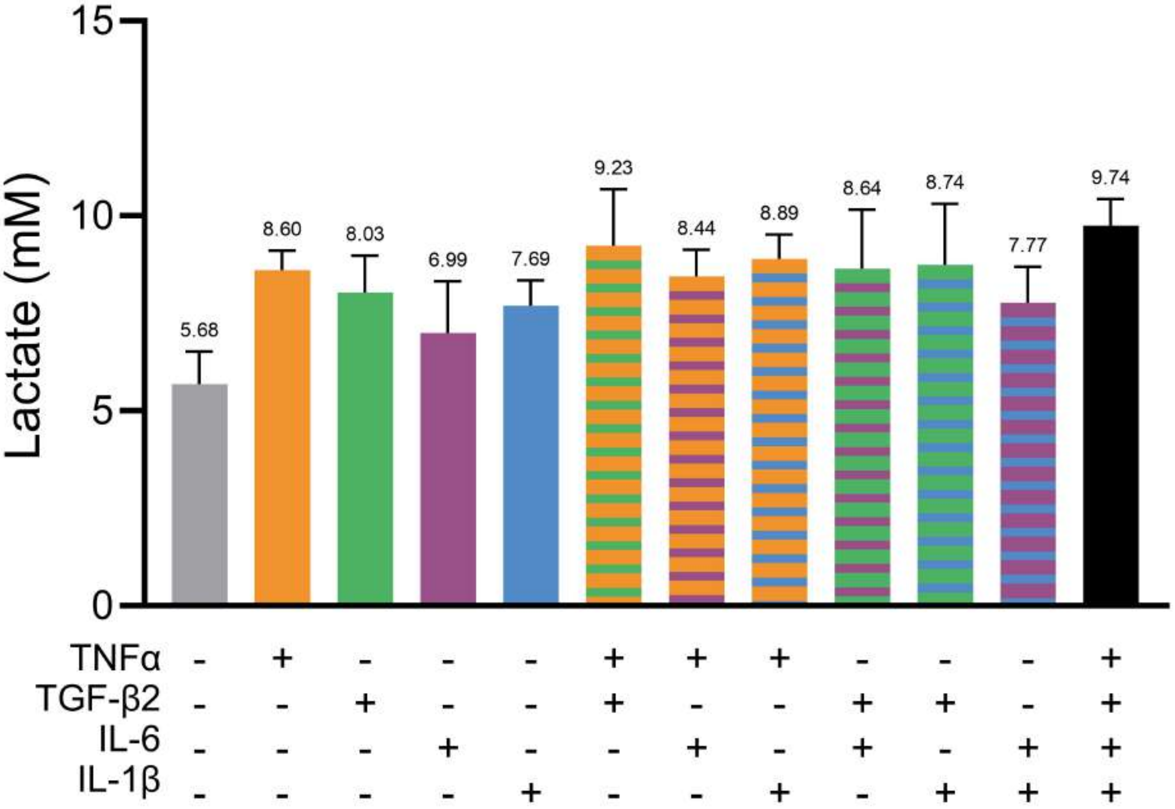

Supplementary figure 1. Cytokines have combinatorial effects on lactate production. The concentration of lactate in the cell culture medium of fully differentiated ARPE-19 cells, measured 24 hours after treatment with different combinations of 20 ng/mL cytokines as denoted (n = 3 independent experiments).

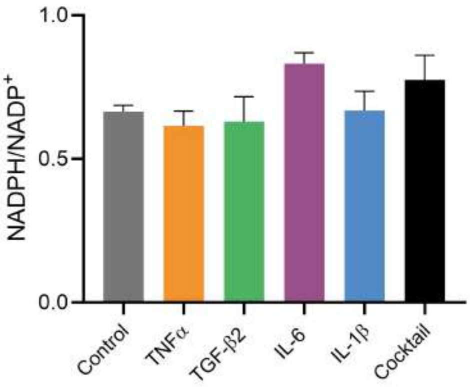

Supplementary figure 2. Cytokines do not alter the NADPH/NADP+ ratio in ARPE-19 cells. NADPH/NADP+ ratio in ARPE-19 cells was calculated post 24-hour cytokine treatment by separately extracting and assaying reduced and oxidized nicotinamide currency metabolites using a cycling method (n = 5 independent experiments) (refer to methods section).

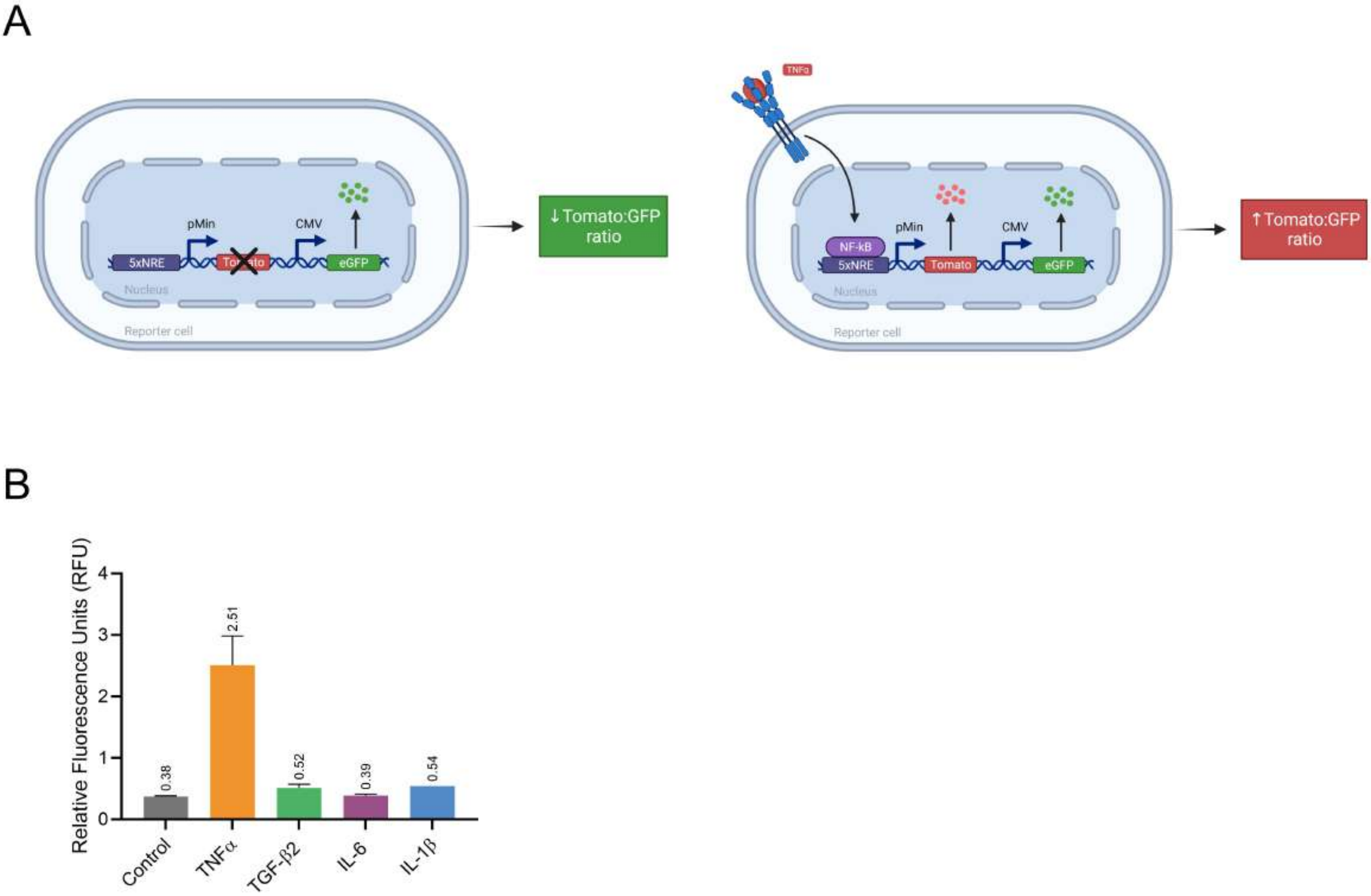

Supplementary figure 3. NF-κB activation in ARPE-19 cells is most potently induced by TNFα. (A) Diagram of the 5XNRE dual fluorescence reporter system. eGFP is basally expressed under the control of a CMV promoter. The activation of NF-κB triggers the expression of Tomato red fluorescent protein, leading to an increase in Tomato:GFP ratio. (B) Tomato:GFP ratios in 5XNRE ARPE-19 cells measured following 24-hour treatment with 20 ng/mL cytokines. Error bars show mean ± SEM. N = 1 independent experiment, performed in triplicate.

Supplementary figure: Uncropped western blots from Figure 4A

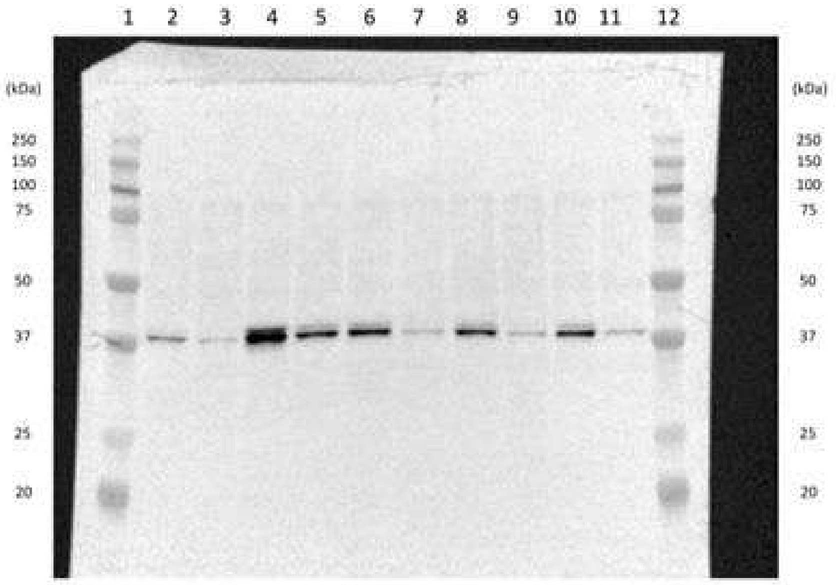

F4C phospho-ERK. Western blot analysis of phospho-ERK in protein extracts from ARPE-19 cells. All 12 lanes were used in Figure 4C. Lanes contain the following: 1 = molecular mass markers, 2 = control, 3 = PD 98059, 4 = TNFα, 5 = PD 98059 + TNFα, 6 = TGF-β2, 7 = PD 98059 + TGF-β2, 8 = IL-6, 9 = PD 98059 + IL-6, 10 = IL-1β, 11 = PD 98059 + IL-1β, 12 = molecular mass markers.

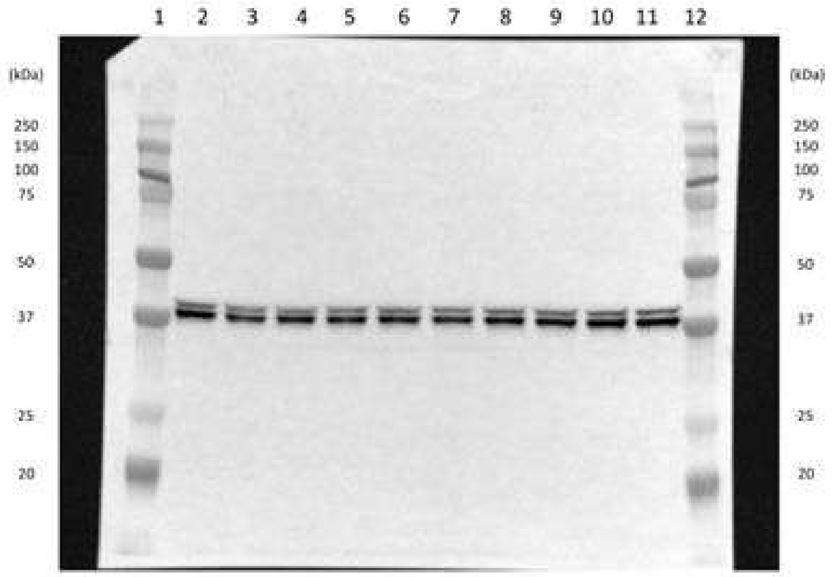

F4C ERK. Western blot analysis of ERK in protein extracts from ARPE-19 cells. All 12 lanes were used in Figure 4C. Lanes contain the following: 1 = molecular mass markers, 2 = control, 3 = PD 98059, 4 = TNFα, 5 = PD 98059 + TNFα, 6 = TGF-β2, 7 = PD 98059 + TGF-β2, 8 = IL-6, 9 = PD 98059 + IL-6, 10 = IL-1β, 11 = PD 98059 + IL-1β, 12 = molecular mass markers.

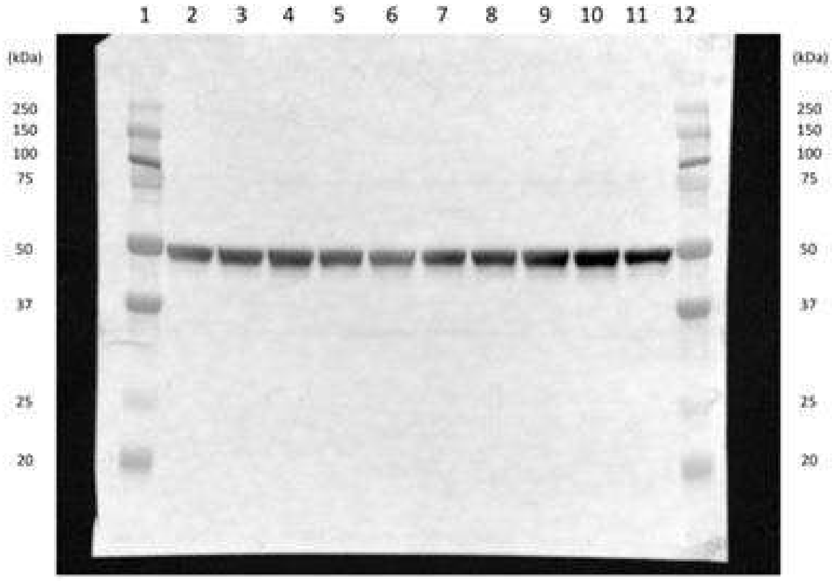

F4C α-Tubulin. Western blot analysis of α-Tubulin in protein extracts from ARPE-19 cells. All 12 lanes were used in Figure 4C. Lanes contain the following: 1 = molecular mass markers, 2 = control, 3 = PD 98059, 4 = TNFα, 5 = PD 98059 + TNFα, 6 = TGF-β2, 7 = PD 98059 + TGF-β2, 8 = IL-6, 9 = PD 98059 + IL-6, 10 = IL-1β, 11 = PD 98059 + IL-1β, 12 = molecular mass markers.

Supplementary figure: Uncropped western blots from Figure 4C

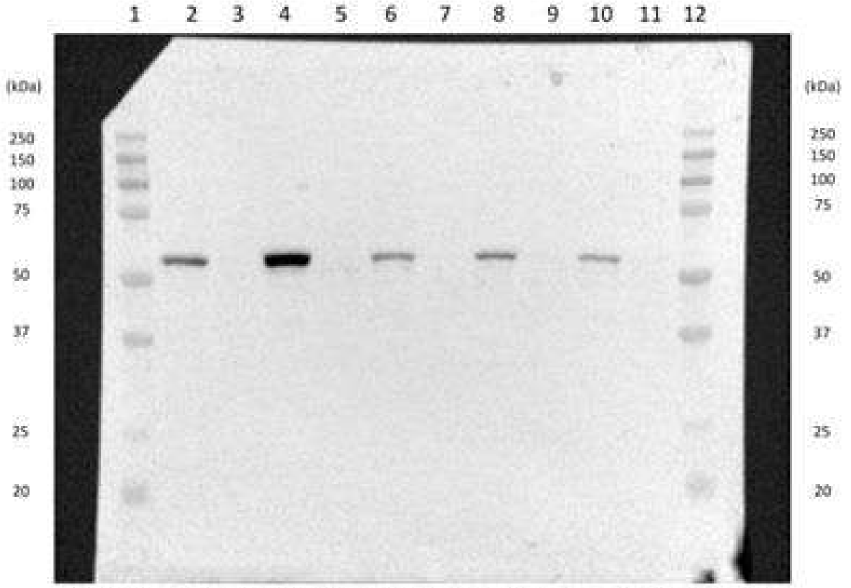

F4A phospho-AKT. Western blot analysis of phospho-AKT in protein extracts from ARPE-19 cells. All 12 lanes were used in Figure 4A. Lanes contain the following: 1 = molecular mass markers, 2 = control, 3 = MK 2206, 4 = TNFα, 5 = MK 2206 + TNFα, 6 = TGF-β2, 7 = MK 2206 + TGF-β2, 8 = IL-6, 9 = MK 2206 + IL-6, 10 = IL-1β, 11 = MK 2206 + IL-1β, 12 = molecular mass markers.

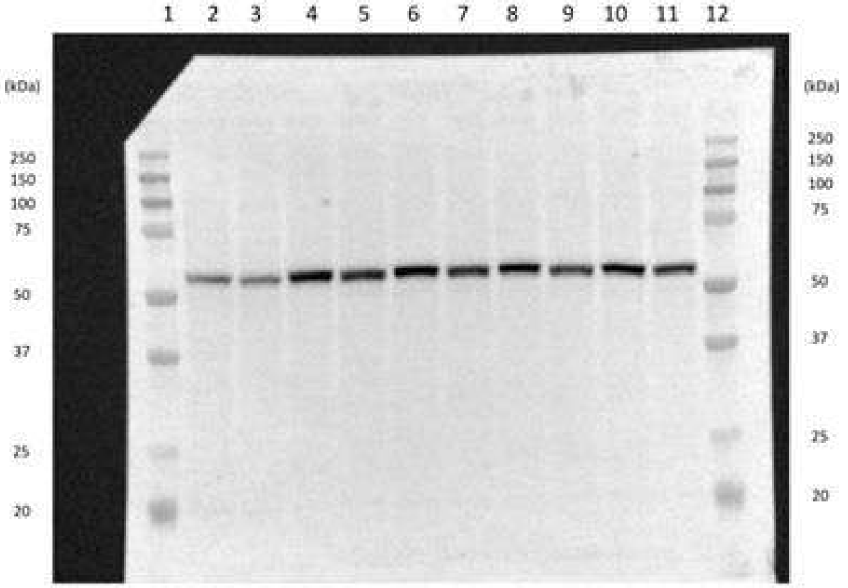

F4A AKT. Western blot analysis of AKT in protein extracts from ARPE-19 cells. All 12 lanes were used in Figure 4A. Lanes contain the following: 1 = molecular mass markers, 2 = control, 3 = MK 2206, 4 = TNFα, 5 = MK 2206 + TNFα, 6 = TGF-β2, 7 = MK 2206 + TGF-β2, 8 = IL-6, 9 = MK 2206 + IL-6, 10 = IL-1β, 11 = MK 2206 + IL-1β, 12 = molecular mass markers.

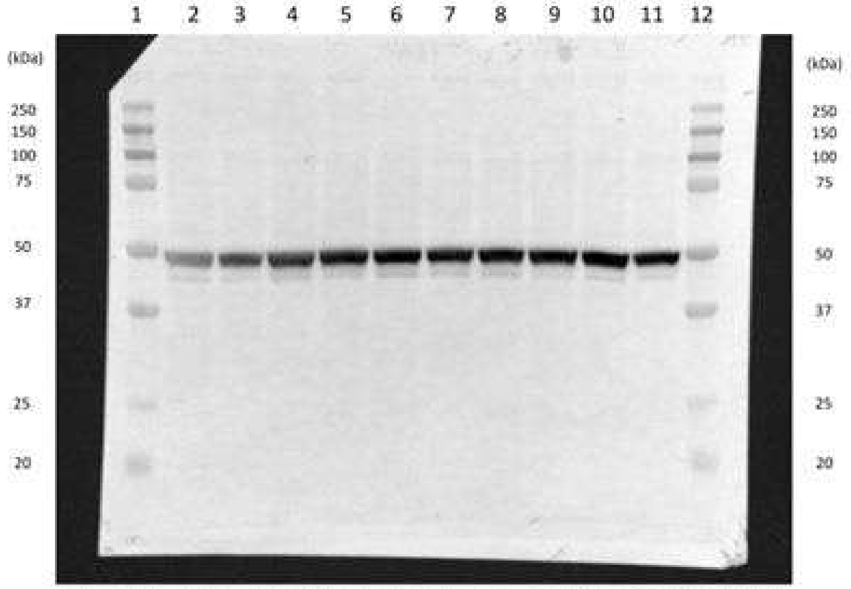

F4A α-Tubulin. Western blot analysis of α-Tubulin in protein extracts from ARPE-19 cells. All 12 lanes were used in Figure 4A. Lanes contain the following: 1 = molecular mass markers, 2 = control, 3 = MK 2206, 4 = TNFα, 5 = MK 2206 + TNFα, 6 = TGF-β2, 7 = MK 2206 + TGF-β2, 8 = IL-6, 9 = MK 2206 + IL-6, 10 = IL-1β, 11 = MK 2206 + IL-1β, 12 = molecular mass markers.
